# Supplementary material for: Induction of Apoptosis via Inactivating PI3K/AKT Pathway in Colorectal Cancer Cells Using Aged Chinese Hakka Stir-Fried Green Tea Extract
Source: Molecules. 2022 Nov 27;27(23):8272. doi: 10.3390/molecules27238272 (PMC9737789; doi:10.3390/molecules27238272)
Supplement: Supplementary file 1 [file molecules-27-08272-s001.zip › molecules-2042465-supplementary.pdf]

Supporting Information

Induction of apoptosis via inactivating PI3K/AKT pathway in colorectal cancer cells using aged Chinese Hakka stir-fried green tea extract

Xinyue Zhang[1,2 †], Haiying Huang[3 †], Shili Sun[2], Dongli Li[1,4], Lingli Sun[2], Qiuhua Li[2], Ruohong Chen[2], Xingfei Lai[2], Zhenbiao Zhang[2], Xi Zheng[1,4], Wing-Leung Wong[1,4,*], Shuai Wen[2,*]

[1] School of Biotechnology and Health Sciences, Wuyi University, Jiangmen 529020, China

[2] Tea Research Institute, Guangdong Key Laboratory of Tea Resources Innovation & Utilization / Guangdong Academy of Agricultural Sciences, Guangzhou 510640, China

[3] Tea Research Institute, Meizhou Academy of Agriculture and Forestry Sciences, Meizhou 514071, China

[4] International Healthcare Innovation Institute (Jiangmen), Jiangmen 529040, China

[†] These authors contributed equally to this work.

* Correspondence: wingleung@wyu.edu.cn (W.-L.W.); wyu2017ws@163.com (S.W.)

(A) HPLC analysis of standard compounds for the verification of the extracted compounds from aged Chinese green tea (HSGT)

A list of standard compounds purchased from commercial:
Gallic acid: **GA**; Gallocatechin: **GC**; Epigallocatechin: **EGC**; Catechin: **C**; Caffeine: **CAFF**; Epicatechin: **EC**; Epigallocatechin-3-gallate: **EGCG**; Gallocatechin-3-gallate: **GCG**; Epicatechin-3-gallate: **ECG**; Catechin gallate: **CG**

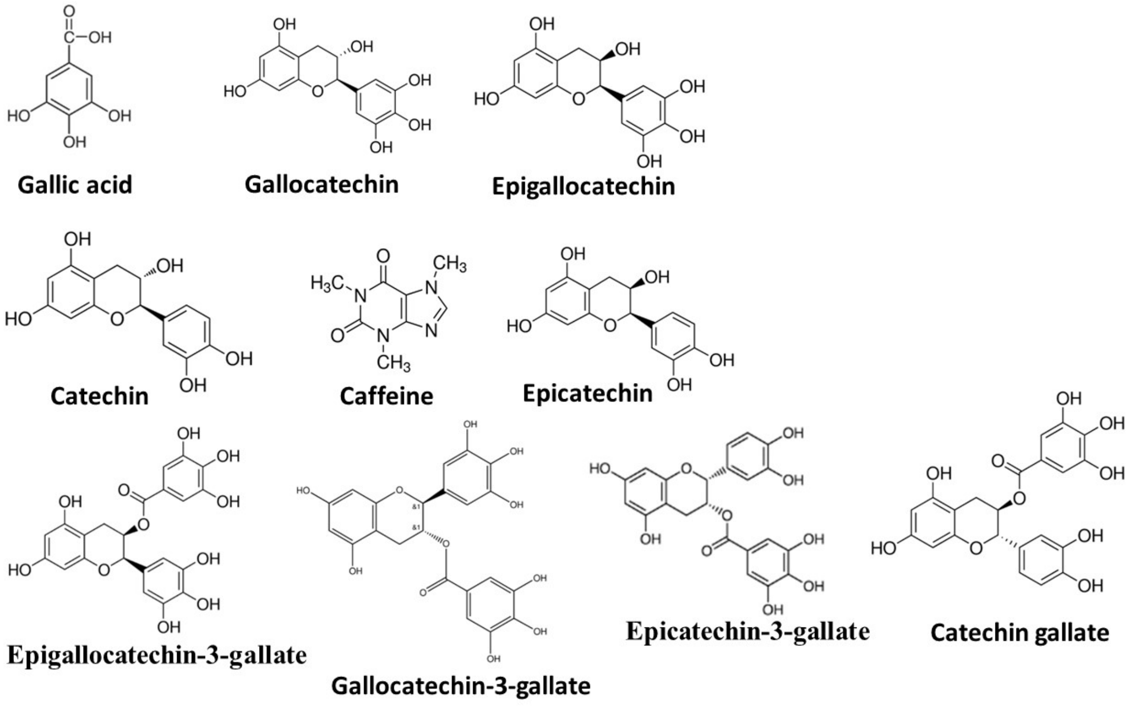

HPLC analytical results for a mixture of the standards:

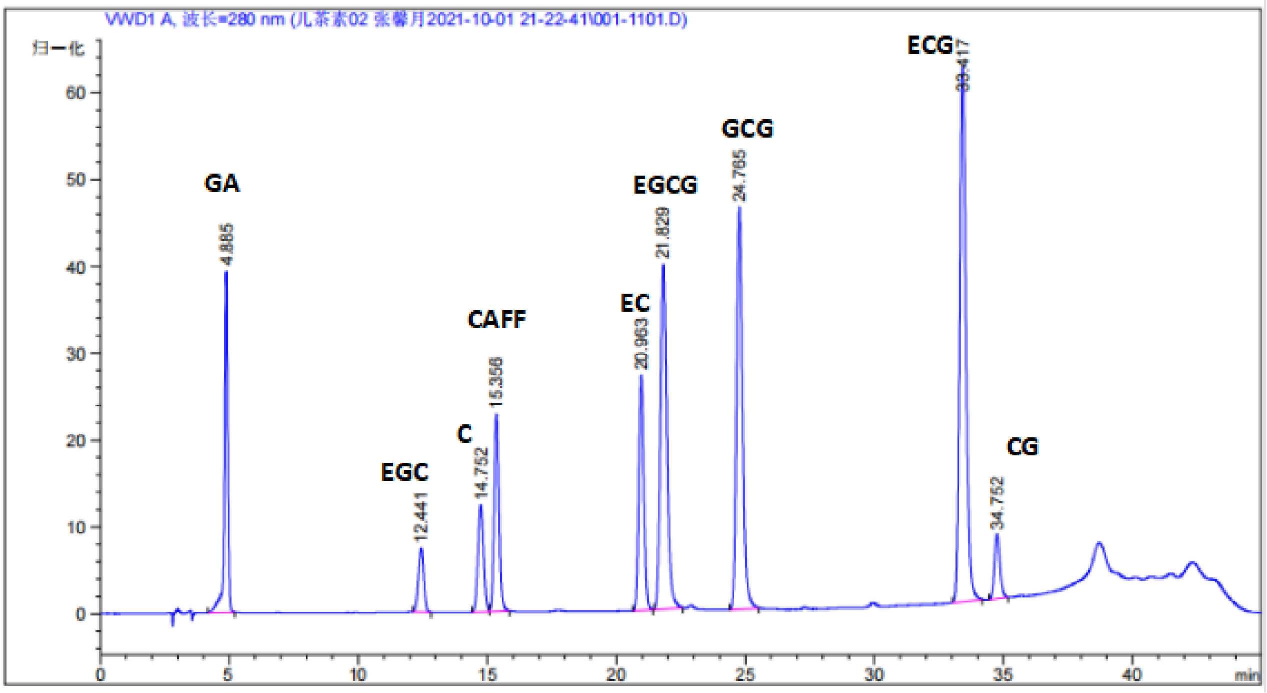

Figure S1: A HPLC chromatogram of a mixture of the standard compounds (HPLC detection wavelength: 280 nm). The compound corresponding to its retention time is labelled and summarized in the Table below.

Standard compound	Retention time (min)
**GA**	4.885
**GC**	7.514
**EGC**	12.441
**C**	14.752
**CAFF**	15.356
**EC**	20.963
**EGCG**	21.829
**GCG**	24.765
**ECG**	33.417
**CG**	34.752

(B) Qualitative and quantitative analysis of the compounds extracted from aged Chinese green tea (HSGT) with HPLC using external standard method

1. Analysis for the extract from tea sample: **03Y**

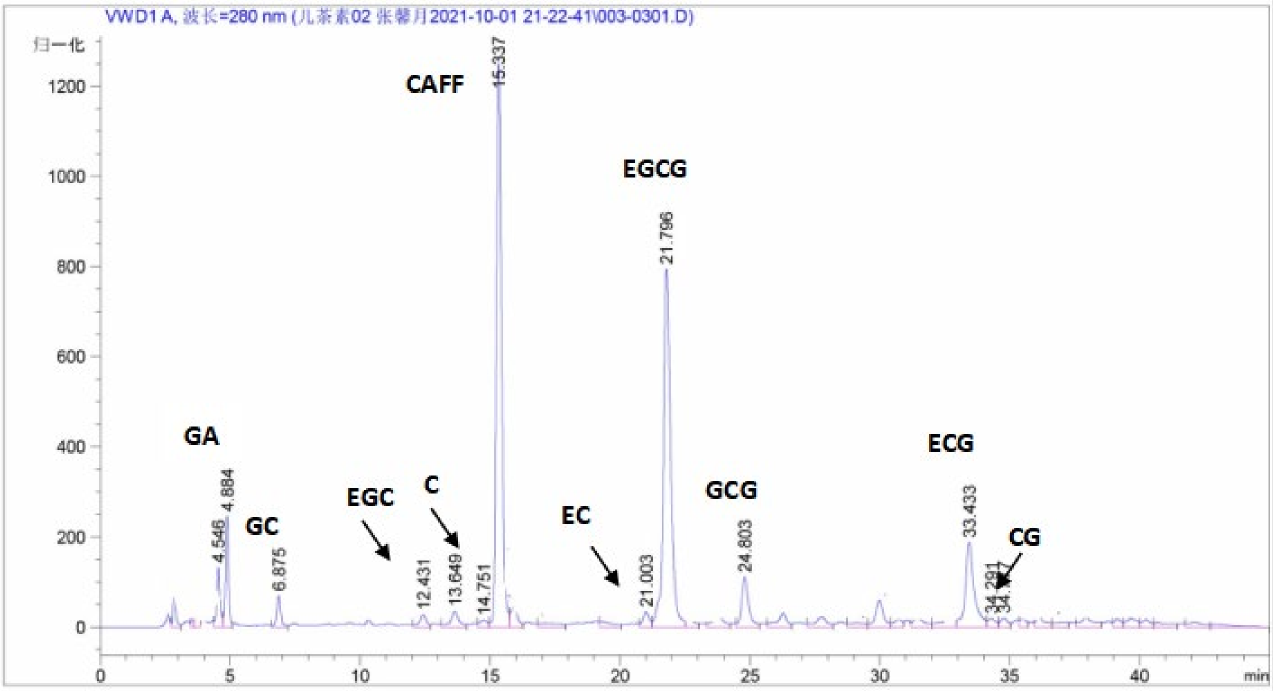

Figure S2: Compounds identified including: **CG**, catechin gallate; **ECG**, Epicatechin-3-gallate; **GCG**, gallocatechin-3-gallate; **EGCG**, epigallocatechin-3-gallate; **EC**, epicatechin; **C**, catechin; **GC**, gallocatechin; **EGC**, epigallocatechin; **GA**, gallic acid; **CAFF**, caffeine.

2. Analysis for the extract from tea sample: **07Y**

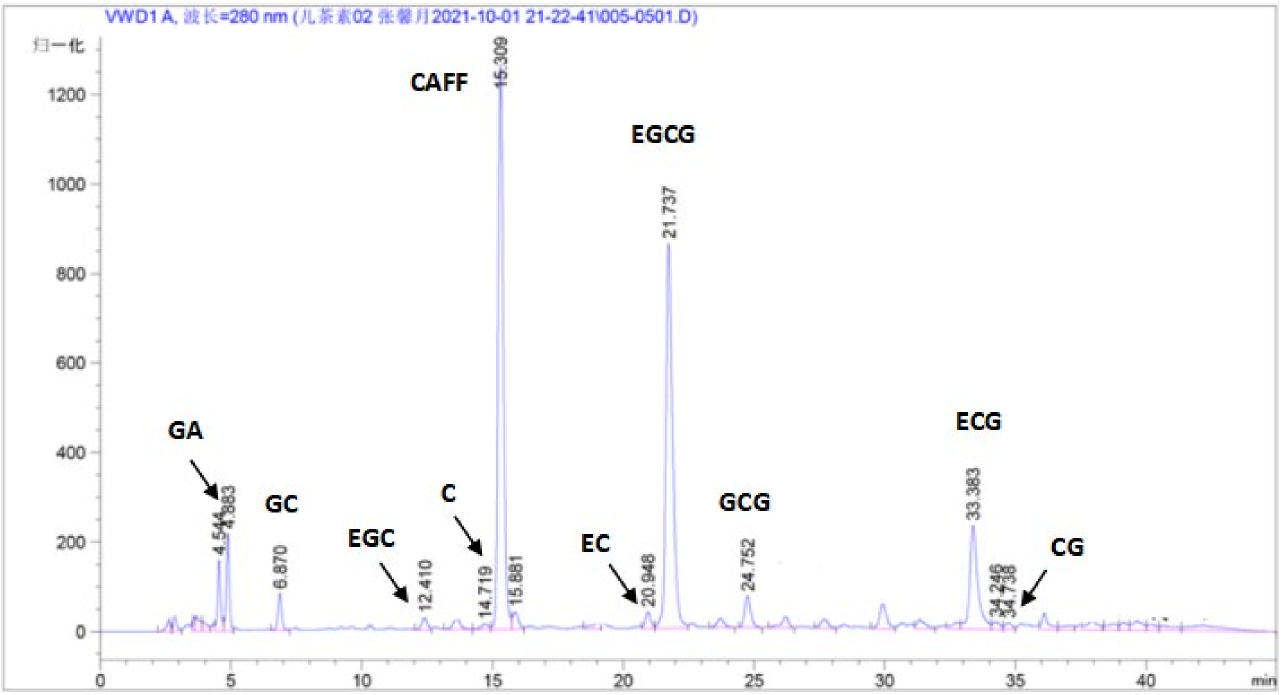

Figure S3: Compounds identified including: **CG**, catechin gallate; **ECG**, Epicatechin-3-gallate; **GCG**, gallocatechin-3-gallate; **EGCG**, epigallocatechin-3-gallate; **EC**, epicatechin; **C**, catechin; **GC**, gallocatechin; **EGC**, epigallocatechin; **GA**, gallic acid; **CAFF**, caffeine.

3. Analysis for the extract from tea sample: **11Y**

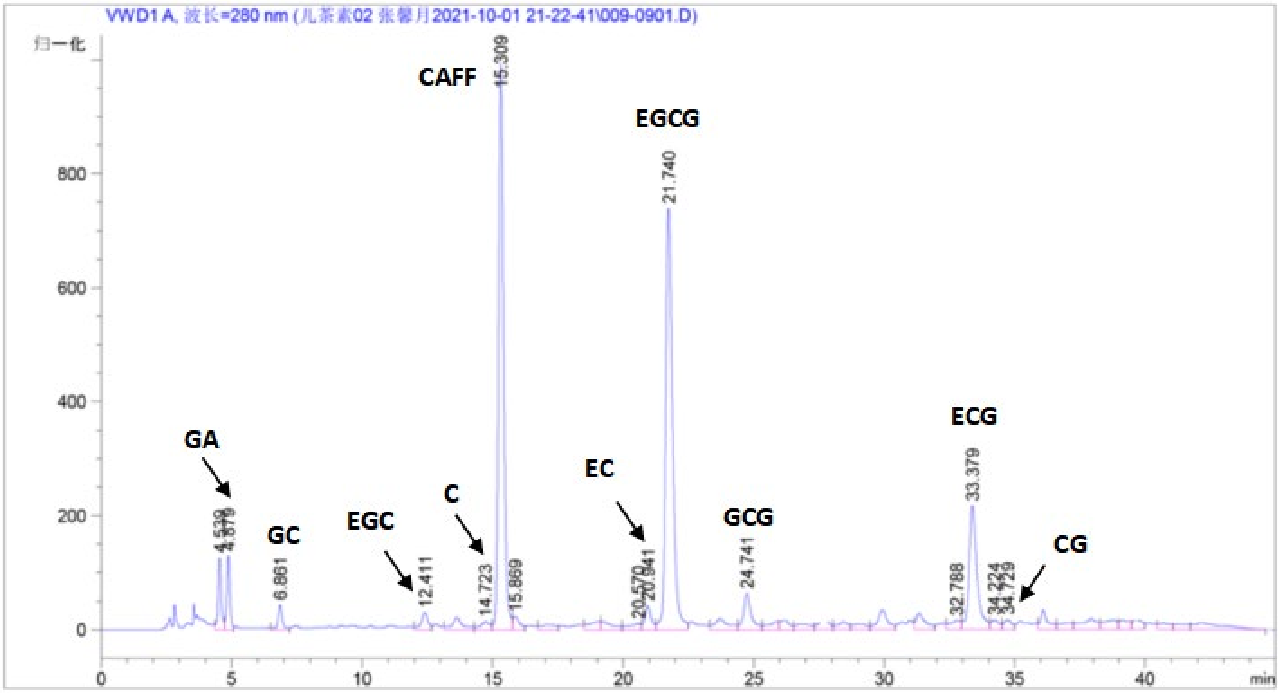

Figure S4: Compounds identified including: **CG**, catechin gallate; **ECG**, Epicatechin-3-gallate; **GCG**, gallocatechin-3-gallate; **EGCG**, epigallocatechin-3-gallate; **EC**, epicatechin; **C**, catechin; **GC**, gallocatechin; **EGC**, epigallocatechin; **GA**, gallic acid; **CAFF**, caffeine.

4. Analysis for the extract from tea sample: **15Y**

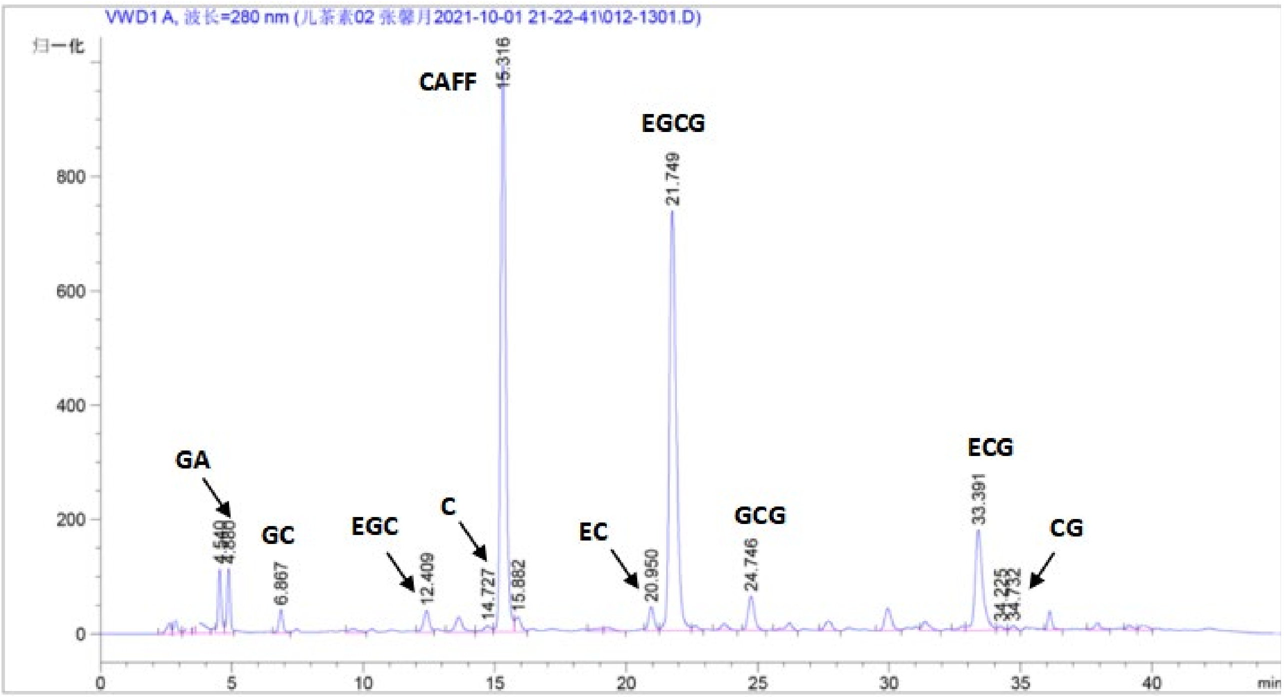

Figure S5: Compounds identified including: **CG**, catechin gallate; **ECG**, Epicatechin-3-gallate; **GCG**, gallocatechin-3-gallate; **EGCG**, epigallocatechin-3-gallate; **EC**, epicatechin; **C**, catechin; **GC**, gallocatechin; **EGC**, epigallocatechin; **GA**, gallic acid; **CAFF**, caffeine.

5. Analysis for the extract from tea sample: **19Y**

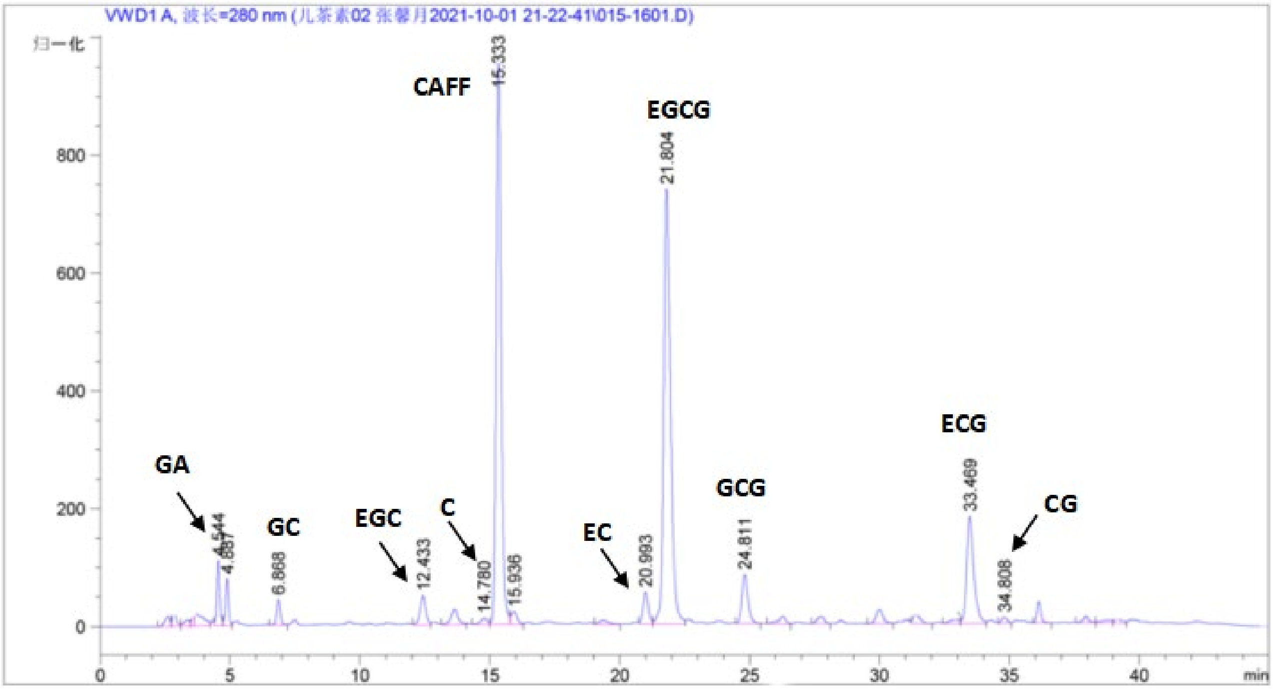

Figure S6: Compounds identified including: **CG**, catechin gallate; **ECG**, Epicatechin-3-gallate; **GCG**, gallocatechin-3-gallate; **EGCG**, epigallocatechin-3-gallate; **EC**, epicatechin; **C**, catechin; **GC**, gallocatechin; **EGC**, epigallocatechin; **GA**, gallic acid; **CAFF**, caffeine.

Table S1. The compounds identified from the extracts of the aged Chinese green tea (HSGT)

Compound/Peak Area	**GA**	**GC**	**EGC**	**C**	**CAFF**	**EC**	**EGCG**	**GCG**	**ECG**	**CG**
Retention Time (min)	4.88	7.51	13.05	15.41	16.02	22.38	23.15	26.23	36.00	37.00
Standard mixture 1	338.54	0	104.24	181.22	301.11	367.07	683.22	758.56	1024.84	105.39
Standard mixture 2	337.76	0	104.48	184.31	299.87	369.82	684.33	748.87	1027.66	109.01
**03Y-1**	1989.29	669.85	430.59	359.61	17552.30	538.93	15592.00	2035.02	4381.74	361.99
**03Y-2**	2024.96	755.60	498.01	317.40	17457.10	566.68	15413.00	2101.71	4452.00	403.33
**03Y-3**	1905.78	674.58	373.01	292.38	16406.30	407.76	14168.50	1684.76	3684.00	156.92
**07Y-1**	1781.31	866.42	412.85	245.23	17363.10	534.38	15654.70	1137.43	4968.73	273.67
**07Y-2**	1797.10	906.43	402.83	242.58	17489.80	527.47	15451.20	1204.82	4910.65	287.38
**07Y-3**	1755.44	889.28	435.00	294.38	16887.30	579.49	15153.00	1327.62	4578.22	247.20
**11Y-1**	1300.96	591.46	597.70	344.70	16503.80	772.28	16537.40	1432.15	5254.98	269.38
**11Y-2**	1073.88	517.24	547.85	347.89	13690.70	720.56	13506.10	1339.82	4533.44	366.64
**11Y-3**	1167.52	525.64	536.31	299.88	14789.20	686.25	14853.50	1269.57	4675.50	227.66
**15Y-1**	1021.42	501.54	681.36	277.41	14719.90	731.77	14915.20	1199.43	3923.16	126.56
**15Y-2**	937.72	445.50	589.87	216.09	13558.70	614.45	13324.00	966.42	3542.82	115.71
**15Y-3**	1113.49	551.73	744.71	300.36	16228.70	801.78	16488.20	1347.79	4342.29	141.71
**19Y-1**	674.92	459.86	794.61	229.22	13440.10	831.37	14164.20	1445.18	3691.57	174.80
**19Y-2**	652.98	439.22	748.73	212.52	12902.20	780.80	13388.90	1370.18	3486.25	149.92
**19Y-3**	672.27	499.59	787.00	245.46	13168.70	798.72	14008.40	1667.30	3672.93	193.24

Table S2. The content of each compound determined from the extracts of the aged Chinese green tea (HSGT)

Sample/Content (mg/g)	**GA**	**CAFF**	**CG**	**ECG**	**GCG**	**EGCG**	**EC**	**C**	**GC**	**EGC**
**03Y-1**	11.49	100.20	2.59	33.48	18.50	151.15	9.76	7.03	69.02	37.40
**03Y-2**	10.85	92.42	2.69	31.55	17.72	138.59	9.54	5.77	72.27	39.90
**03Y-3**	10.91	92.92	1.00	27.91	15.21	136.51	7.24	5.70	69.02	32.37
Average	10.88	92.67	2.64	29.73	16.47	137.55	9.65	5.74	69.02	38.65
SD	0.03	0.25	0.05	0.96	0.39	1.04	0.11	0.03	0.00	1.25
**07Y-1**	9.20	88.98	1.71	34.11	9.29	140.08	8.69	4.34	80.30	32.25
**07Y-2**	9.96	96.15	1.94	36.16	10.55	144.26	9.19	4.61	90.13	33.79
**07Y-3**	9.62	91.91	1.62	33.37	11.51	140.16	10.04	5.51	87.57	36.03
Average	9.79	90.45	1.67	33.74	10.45	141.79	9.31	4.48	86.00	33.02
SD	0.17	1.47	0.04	0.37	0.48	0.04	0.25	0.13	1.28	0.77
**11Y-1**	6.30	80.69	1.79	38.50	12.47	153.46	13.57	6.46	58.31	49.16
**11Y-2**	7.05	75.95	2.51	33.14	11.65	151.77	12.62	6.51	50.86	45.12
**11Y-3**	5.85	75.74	1.51	34.75	11.22	136.25	12.20	5.72	52.55	44.94
Average	6.40	75.85	1.65	33.94	11.44	146.77	12.41	6.49	51.71	45.03
SD	0.22	0.10	0.14	0.81	0.21	0.85	0.21	0.02	0.85	0.09
**15Y-1**	5.55	81.64	0.75	29.16	10.62	140.85	13.05	5.31	50.19	56.77
**15Y-2**	5.12	90.05	0.68	26.60	8.65	127.38	11.02	4.21	45.02	49.90
**15Y-3**	5.94	87.93	0.84	31.51	11.64	125.42	13.99	5.60	53.92	60.41
Average	5.54	88.99	0.76	30.34	11.13	131.21	13.52	5.46	52.05	58.59
SD	0.33	1.06	0.04	1.18	0.51	0.98	0.47	0.14	1.87	1.82
**19Y-1**	3.45	72.63	1.09	26.76	12.48	130.57	14.52	4.30	44.86	64.36
**19Y-2**	3.37	70.63	0.92	25.60	12.00	125.19	13.80	4.05	43.41	61.56
**19Y-3**	3.51	72.61	1.25	27.17	14.70	131.82	14.22	4.70	49.78	65.08
Average	3.45	71.96	1.08	26.51	12.24	131.20	14.37	4.35	44.13	63.67
SD	0.03	0.01	0.08	0.21	0.24	0.63	0.15	0.26	0.73	0.36

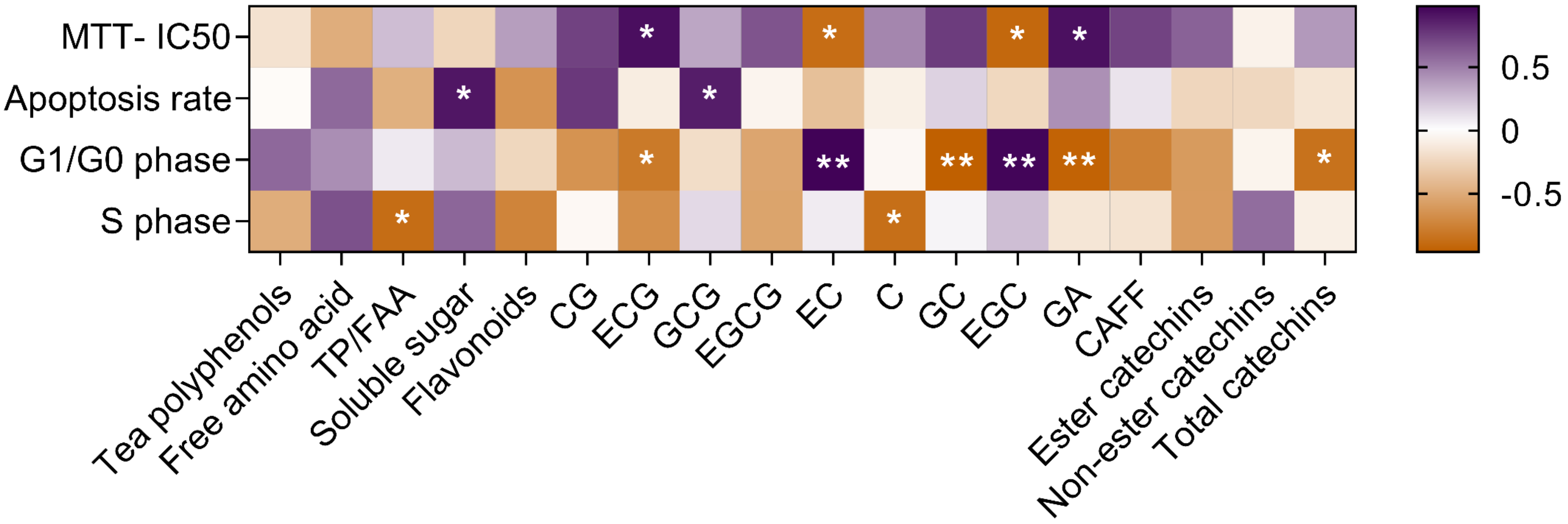

Figure S7. Correlation heatmap analysis of HSGTs extract active ingredients with HT-29 cell-related indexes. The HSGTs extracts were obtained from five different tea samples including **03Y**, **07Y**, **11Y**, **15Y** and **19Y**. The HT-29 cell-related indexes include cell proliferation (MTT-IC_{50}, at 48 h), cell apoptosis (Apoptosis rate, at 48 h 0.2 mg/mL), and cell cycle blockade (G1/G0 phase, S phase, at 48 h 0.2 mg/mL). Pearson correlation analysis was performed. Purple and brown squares indicated positively and negatively correlation, respectively. The *p*-value was calculated from the one-tailed option with a confidence interval of 95%. The statistically significant correlation was showed as $^*p < 0.05$ and $^{**}p < 0.01$.
